# Micro-livestock in smallholder farming systems: the role, challenges and opportunities for cavies in South Kivu, eastern DR Congo

**DOI:** 10.1007/s11250-019-02112-9

**Published:** 2019-11-22

**Authors:** Charlotte J. Klapwijk, Marc Schut, Piet J. A. van Asten, Bernard Vanlauwe, Ken E. Giller, Katrien Descheemaeker

**Affiliations:** 1 International Institute of Tropical Agriculture (IITA, Bukavu, Democratic Republic of Congo; 2 Plant Production Systems Group, Wageningen University & Research (WUR), P.O. Box 430, 6700 AK Wageningen, The Netherlands; 3 International Institute of Tropical Agriculture (IITA), Kigali, Rwanda; 4 Knowledge, Technology and Innovation Group, Wageningen University & Research (WUR), P.O. Box 8130, 6700 EW Wageningen, The Netherlands; 5 International Institute of Tropical Agriculture (IITA), Kampala, Uganda; 6 Olam International Ltd., Kampala, Uganda; 7 International Institute of Tropical Agriculture (IITA), Nairobi, Kenya

**Keywords:** DR Congo, *Cavia porcellus*, Micro-livestock, Smallholder livelihoods

## Abstract

Livestock play multiple roles for smallholder farmers in sub-Saharan Africa. Mixed crop-livestock systems are common in South Kivu, eastern DR Congo, but herd sizes are small and numbers of large livestock (i.e. cattle) have declined, due to high population density, recent conflicts and extreme poverty. Over half of the farmers keep cavies, a type of micro-livestock fitting the circumstances of smallholders and a valuable asset especially for the poorest households. To characterize cavy husbandry practices, detailed monthly on-farm data on cavy numbers, weights, herd dynamics and feeding practices were collected over 15 months and from households in two contrasting sites in South Kivu. Cavy herds contained on average 10 animals and strongly varied in size over time and between households. The main reasons for keeping cavies were meat consumption, especially for children, and the opportunity to generate petty cash. A large difference was observed in adult cavy live weights between the sites (an average of 0.6 and 1.0 kg per animal in Kabamba and Lurhala, respectively) and attributed to differences in cavy husbandry and genetics. In both sites, quantities of fresh fodder on offer were larger than fodder demand by 50–100%, but no correlation was found between amount of fodder on offer and cavy weight. Farmers faced several constraints to cavy production, including substantial declines in cavy herd size due to predation or theft and a lack of knowledge regarding breeding and feeding. Hence, the introduction of cages to limit mortality and fodder cultivation to improve feed quality were opportunities for improving cavy production. Overall, micro-livestock present a promising entry-point for development initiatives, also outside DR Congo, because of their potential to decrease poverty and improve human nutrition.

## Introduction

Livestock play multiple important roles for smallholder farmers in sub-Saharan Africa (Herrero et al. 2013; Lammers et al. 2009). They provide animal products for sale or consumption, including manure for crop production (Herrero et al. 2013), are used to invest and save capital and play an important role in socio-cultural events (e.g. marriage). As in many parts of SSA, smallholder farming systems in the eastern province of South Kivu in the Democratic Republic of Congo (DR Congo) are diverse, often with livestock as an integral part of the system (Cox 2012). However, current herd sizes are small due to poverty, pressure on resources and high population density (Cox 2012) and as a consequence of recent violent conflicts (Ouma and Birachi 2011; Maass et al. 2012). In this context of dire poverty and insecurity, approximately half of the smallholder farmers keep cavies (also known as guinea pigs—*Cavia porcellus*) (Maass et al. 2010, 2014) for meat production and to generate income (Meutchieye et al. 2013). Cavies are a type of micro-livestock: inherently small species of livestock (i.e. poultry, rabbits, rodents, etc.) with a multitude of benefits that fit the circumstances of smallholder farmers (NRC 1991; Desiere et al. 2015).

In the early 20th century, cavies were first kept in convents by European Catholic missionaries in DR Congo (Maass et al. 2014). Local people working at convents took the animals home for their children, but many people considered cavies as a sort of rat, not welcome inside the house (Maass et al. 2014). Widespread famine and malnutrition caused by successive conflicts in the 1980s resulted in non-governmental development organisations recommending a mix of tomato concentrate, cola and cavy blood as medicine to address anaemia in children (Maass et al. 2014), which enhanced the acceptance of cavies as livestock. The recurrence of wars over the past decades is a major factor that explains the current importance of cavies for smallholder farmers. Large livestock are usually the target of looting, while the small cash value of cavies results in less risk of theft, and they can easily be hidden or carried when fleeing (Cox 2012; Desiere et al. 2015). Due to their small size, they require little investment (in e.g. feed and space), and their rapid rate of reproduction makes cavy herds resilient to shocks (Bindelle and Picron 2012; Lammers et al. 2009).

Estimates of the current cavy population in South Kivu are uncertain, but indicate at least 500,000 cavies (Meutchieye et al. 2013). Cavies are often the only type of livestock owned by the poorest families (e.g. female-headed households or widows). In male-headed households, cavies are usually the responsibility of women or youth (Maass et al. 2014) and are therefore a relatively accessible source of animal protein and petty cash for those household members. In South Kivu, nearly all children and about two thirds of women and men consume cavies (Meutchieye et al. 2013), thus improving their diet quality (Murphy and Allen 2003). Despite being generally accepted as livestock, cavies are usually associated with poverty (Maass et al. 2010) and the consumption of cavy meat is often referred to be for children.

Although the importance of cavies within the smallholder farming systems of South Kivu is evident, current cavy husbandry practices, such as feeding, have not been described and production has not been quantified. Furthermore, constraints and benefits of keeping cavies have not been studied in detail. The main objective of this research was therefore to characterize smallholder cavy production systems in South Kivu, as well as the importance of cavies for meat consumption and income. Specific objectives were to assess at farm-level: (i) current cavy production in terms of weight and number of cavies per household, (ii) feeding practices (i.e. fodder types and their quantity on offer and refused) and (iii) consumption and sales of cavies.

## Methods

### Research sites

**Fig. 1:**
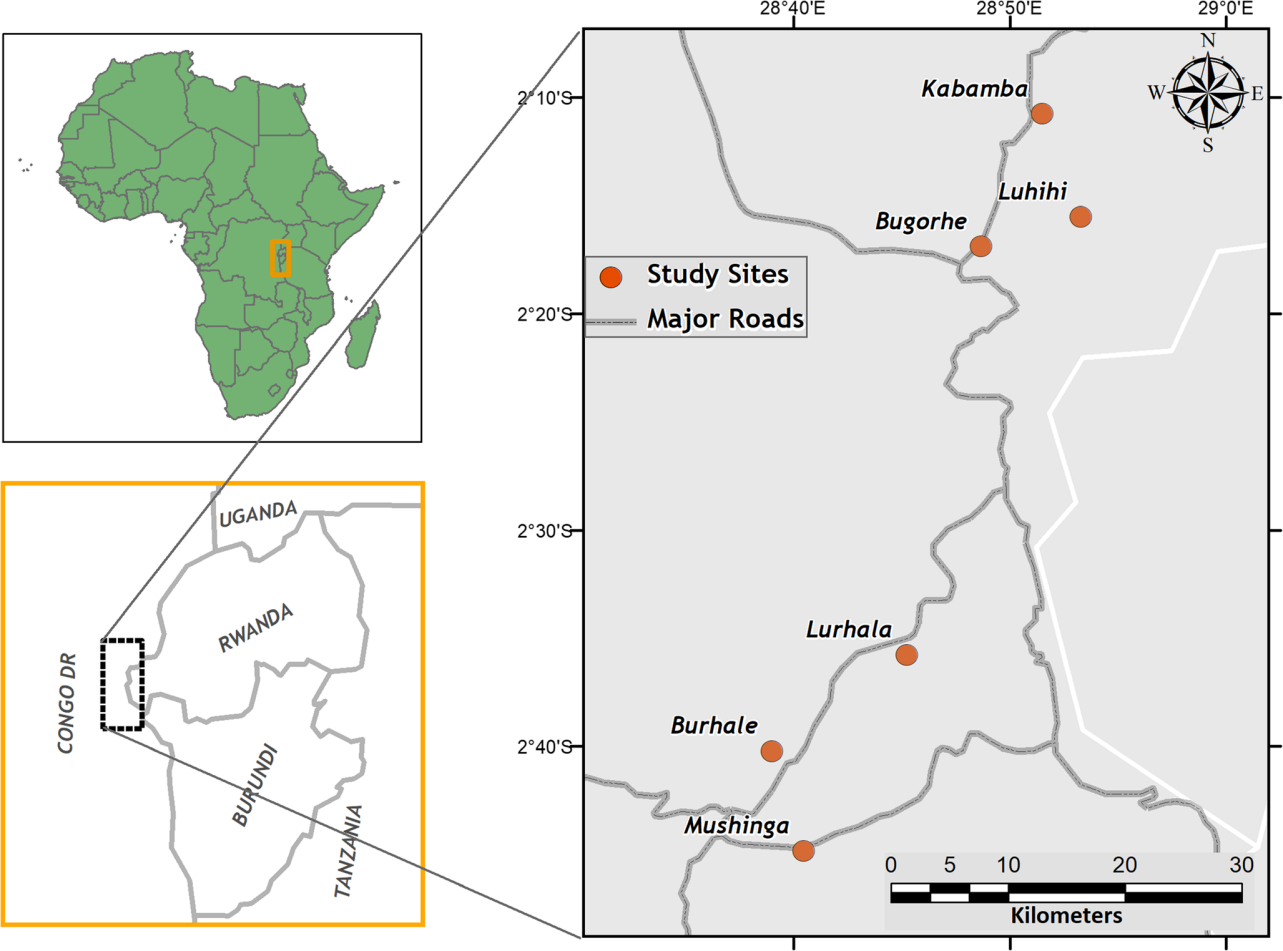
Map of the six sites where the focus group discussions were conducted. Sites in the North belonged to Kabare territory and in the South to Walungu territory

The research was conducted in two contrasting territories in South Kivu, Kabare and Walungu, with the former characterized by easier market access and better soil fertility compared to the latter (Ouma and Birachi 2011). The research started with focus group discussions (FGD) in six representative sites (three in each territory) and then zoomed in on two of the six sites for the remaining data collection (i.e. household survey, on-farm measurements) (Fig. 1). These main research sites comprised ‘groupements’, i.e. a cluster of villages: (i) Kabamba, in Kabare territory, and (ii) Lurhala, in Walungu territory (Table 1). Elevation was 1600 m a.s.l. in Kabamba and 2000 m a.s.l. in Lurhala. For both sites, annual precipitation ranged from 1500 to 1800 mm, mean temperature was 21 °C and population density was > 250 people km^−2^ (Pypers et al. 2011). Farm sizes were relatively small (< 2 ha) and farming systems typically integrated livestock and crops, usually intercropping several crops in one field. The area had two rainy seasons, from September to December and from January to June, and a dry season in July and August.

**Table 1:** Main characteristics of the research sites

Variables	Kabamba (Kabare territory)	Lurhala (Walungu territory)
Average household size^a^	9 ± 3	11 ± 5
Average farm size (ha)^a^	0.77 ± 0.70	1.25 ± 1.09
Food crops^a^	Bush bean, maize, cassava (and taro)	Sweet potato, cassava, taro (and maize)
Cash crops^a^	Cassava, coffee	Banana, maize
Dominant soils^b^	Relatively fertile Humic Nitisols/Ferralsols	Rather infertile Dystric or Humic Nitisols/Ferralsols
Length of growing period (days)^b^	> 325	> 325

^a^Data of participating households (*n* = 27 per site)

^b^Pypers et al. 2011

### Data collection

A mixed methods approach, combining FGDs, a household survey and on-farm measurements, was used. The FGDs and survey were conducted with a representative sample of the population in order to understand the region’s household diversity with respect to livestock keeping. As cavies were particularly important for the poor, purposeful sampling of these households was conducted for the detailed on-farm measurements of cavy husbandry. Price data of three types of micro-livestock (chickens, rabbits and cavies) were collected on the local market near the research sites (i.e. Mugogo and Katana) and in Bukavu, the capital of South Kivu.

#### Focus group discussions

Focus group discussions (in total six) were held in three representative sites per territory. The objective was to gain insights on livestock ownership, in terms of livestock types (micro-livestock and other) and reasons (not) to own these livestock types. The FGDs were organized in cooperation with a local facilitator, who assisted by inviting a representative sample of ten men and ten women farmers of different age groups and resource endowment. The final number of participants varied from 14 to 20 per FGD. The illiteracy rate in the groups ranged between 16 and 57% and illiterate participants were assisted by a colleague or research assistant. The general discussions took place with the whole group, but participants were divided by gender to list and rank the three main reasons for (not) keeping livestock.

#### Household survey

A survey was conducted with 27 households per site to describe general farm characteristics, family composition and household food consumption, as well as livestock keeping. In each site, a local facilitator assisted with the categorization of households into a context-specific typology of ‘poor’, ‘medium’ and ‘better-off’ based on physical resources such as land, livestock ownership and housing quality and provided a list of around 20 households per type. Poor households were considered to own less than 0.5 ha of land, keep only micro-livestock and sometimes a few goats and live in a low-quality house made of mud walls and a grass roof. The facilitator described medium resource-endowed households as owning between 0.5 and 2 ha of land, micro-livestock and some goats, and houses with an iron-sheet roof. The third household category was considered better-off in terms of land area exceeding 2 ha, keeping at least one cow and living in houses with durable walls and roof. A total of 27 livestock-keeping households were selected per site, with a fair representation of male- and female-headed households. For this research on cavies and further research on cows and goats, nine farmers keeping cavies from the poor farm type, nine farmers keeping cows from the better-off type and nine farmers keeping goats (three from each type) were included. This resulted in an unbalanced sampling from a resource-endowment point of view and numbers of farmers slightly differed from the planned numbers due to initial mis-classification on the facilitator’s list (Table 2). Herd sizes were expressed in tropical livestock unit (TLU, 250 kg) for each household, based on a factor 1 for cattle, 0.46 for pig, 0.12 for sheep and goat, 0.01 for chicken and rabbit and 0.004 for cavy.

**Table 2:** Mean (and standard deviation in brackets) of animal number and total herd size (in TLU) per farm type per site

	Kabamba (Kabare territory)			Lurhala (Walungu territory)		
Livestock type	Poor (*n* = 13)	Medium (*n* = 4)	Better-off (*n* = 10)	Poor (*n* = 12)	Medium (*n* = 4)	Better-off (*n* = 11)
Cow	0.2 (0.6)	0.0	1.6 (1.7)	0.3 (0.9)	0.0	1.5 (1.4)
Heifer	0.0	0.0	0.3 (0.5)	0.0	0.3 (0.5)	0.5 (0.9)
Bull	0.0	0.0	0.0	0.2 (0.4)	0.3 (0.5)	0.0
Sheep	0.0	0.0	0.0	0.2 (0.4)	0.5 (1.0)	0.6 (0.9)
Goat	2.8 (4.2)	6.3 (5.6)	4.0 (3.7)	2.1 (1.7)	2.3 (1.9)	2.2 (1.5)
Pig	0.4 (0.7)	0.8 (1.0)	0.7 (1.6)	0.6 (0.9)	1.3 (1.5)	1.7 (3.4)
Chicken	2.8 (4.2)	5.0 (7.4)	7.0 (6.4)	1.8 (2.9)	3.5 (1.9)	5.7 (3.0)
Rabbit	0.2 (0.8)	0.0	0.0	0.8 (2.1)	1.3 (1.9)	1.3 (1.7)
Cavy	6.5 (5.5)	23.5 (20.6)	11.0 (17.2)	5.0 (3.4)	8.8 (6.3)	12.4 (7.7)
Total (TLU)	0.8 (0.9)	1.2 (0.8)	2.7 (2.7)	1.0 (0.9)	1.3 (1.0)	3.1 (2.3)

#### On-farm cavy production and fodder measurements

At each site, the nine cavy-keeping farmers, categorized as “poor”, were invited to participate in the research of which a total of 15 households contributed reliable data (8 in Kabamba and 7 in Lurhala). Weight (in gram) of individual cavies was recorded monthly at each household during 15 consecutive months: from October 2015 until December 2016. Cavies were categorized into one of three categories, including (i) suckling: less than 2 weeks old, not yet eating grass, (ii) young: over 2 weeks old till first pregnancy and (iii) mature: first pregnancy and after. Research assistants noted pregnancy cases during weight measurements. The reasons behind changes in cavy number were also recorded on a monthly basis.

Besides the monthly measurements by research assistants, the 15 households made daily recordings of (i) fodder quantity on offer per fodder type (in kg) and (ii) fodder refusals (either per type or in total—depending on the farmer). These on-farm measurements took place during 20 consecutive months, from May 2015 until December 2016, resulting in over 13,000 observations. Participating households received training and necessary utensils, including a 0–12 kg weighing scale, pencil, eraser and weekly data collection sheets). To gain insight in fodder composition, all recorded fodder types (*n* > 30) were categorized in one of six fodder groups (i) cultivated grass (e.g. *Pennisetum purpureum* Schumach, *Tripsacum laxum* Nash), (ii) naturally growing grasses (e.g. *Setaria sphacelata* (Schum.) Stapf & Hubb., *Hyparrhenia rufa* (Nees) Stapf, *Cynodon aethiopicus* Clayton & Harland), (iii) weeds (e.g. *Tithonia diversifolia* (Hemsl.) A. Gray, *Commelina diffusa* Burm.f., *Galinsoga ciliata* (Rafin.) Blake), (iv) banana plant parts (leaves and pseudo-stem), (v) crop residues (sweet potato, beans, maize, groundnut, sugarcane) and (vi) “others”. The latter category included fodder that was not specified by the farmers and mixtures of leaves, crop residues and various plant parts. Some rare fodders were excluded from the analysis (e.g. avocado leaves occurring twice, reed occurring 14 times).

#### Assessment of animal product consumption

Household consumption of animal products was assessed based on the household survey (see “Household survey”) and on farmer records of 85 consecutive weeks from May 2015 to end of December 2016. The survey provided recall data of the consumption frequency of animal products for four preceding seasons: the long rainy season (Jan–Jun 2015), short rainy season (Sep–Dec 2014), dry season (Jul–Aug 2014) and the previous long rainy season (Jan–Jun 2014). Weekly farmer records allowed the calculation of consumption frequency of 10 animal products, including beef, goat, pork, chicken, cavy, small fish, big fish, eggs, fresh milk and insects. To gain insight in the importance of cavies, the consumption frequency of cavy meat was compared to the consumption frequency of meat in general.

## Results

### Livestock and cavy ownership

Goats, cavies and chickens were owned by 64%, 52% and 50% of the FGD participants, respectively, while less than 30% of participants owned cattle. In Walungu, 42% of participants owned cattle compared with only 15% in Kabare, with a similar trend for sheep (15% vs. 2%) and rabbits (21% vs. 9%). Herds were larger in Lurhala with a mean total herd size of 1.2 TLU compared to 0.95 TLU in Kabamba (Table 2). Gender-related differences in ownership were found in the proportions of participants owning cattle (higher for men) and owning no animals at all (higher for women).

All six focus groups listed the same three reasons to own micro-livestock: to provide (i) food, (ii) cash and (iii) manure. Most farmers linked cash needs to the payment of children’s school fees. Surprisingly, only farmers in Kabare mentioned ‘medicinal’ as a reason to keep cavies and in all cases, women voted this reason into their top three. In two FGDs, men voted ‘gift for marriage/to visitors/to reinforce friendships’ to be among the most important reasons to own micro-livestock. The three main reasons not to own micro-livestock were (i) highly susceptible to diseases (mainly for rabbits) and lack of veterinary products, (ii) easy victims to predation by cats, dogs and bird of prey and (iii) frequent victims of theft (by neighbours). Another reason, voted among the top three by men and women in half of the FGDs was ‘lack of training and knowledge on husbandry and breeding’.

### Cavy production

#### Cavy keeping

Cavies were generally kept in the house or kitchen and were fed crop residues, kitchen waste and collected fodders. Fodder was usually collected along cropping fields and roads and offered on the ground without a feeder. All participating households collected manure of cavies and composted it before applying it to homestead gardens or fields. Main reasons for the absence of cages, as clarified by the farmers, were a lack of resources in terms of money or space, an increased incidence of pests and diseases when animals were caged and the animals’ nature (i.e. cavies prefer to be free).

#### Cavy herd size

**Fig. 2:**
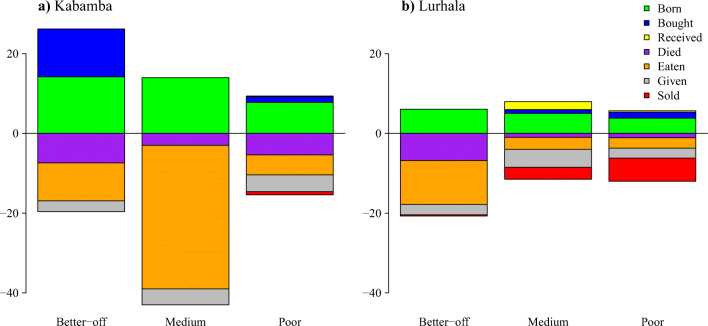
Annual numbers of cavies per site as recorded in the survey

The mean reported cavy herd size was ten animals per household. For the year prior to the household survey, farmers recalled an average of eight cavies born, against five cavies that died (Fig. 2), illustrating the dynamics within a cavy herd.

**Fig. 3:**
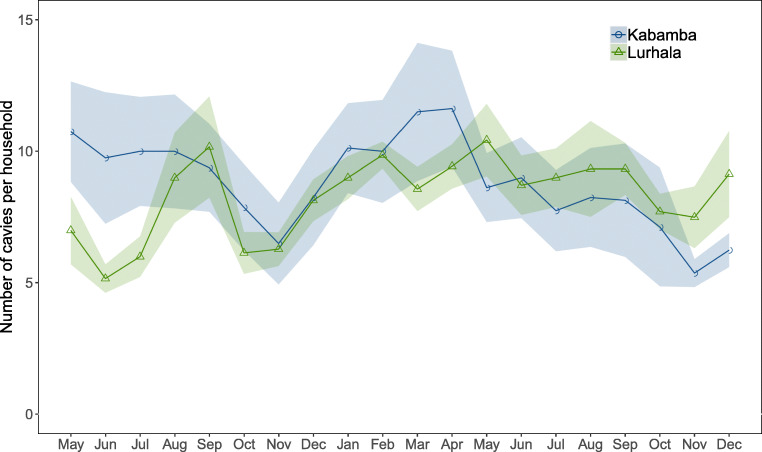
Mean cavy number per household (plus and minus the standard error indicated by the shaded area) in both sites over time

The cavy monitoring data allowed characterizing these dynamics more thoroughly. Cavy herd size per individual household fluctuated strongly and frequently, with a range of 4–17 cavies in Kabamba (mean: 13 cavies) and 8–19 cavies in Lurhala (mean: 11 cavies). During the monitoring period, several farmers lost over half of their animals in the course of 1 month. Consumption and sales of cavies, as well as disasters such as predation by a cat or dog, and theft were common explanations for the reported decreases in animal numbers. Herds also rapidly regained numbers thanks to cavies’ high reproduction rates and their low market price (around 1 USD per mature animal). Despite these dynamics, the mean total number of cavies per household was relatively stable over time in both sites (Fig. 3).

#### Cavy weight

**Fig. 4:**
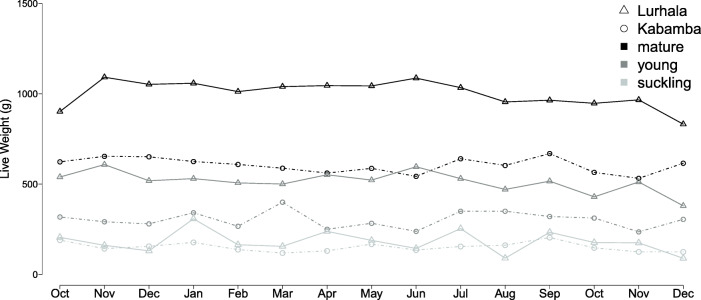
Mean weights of mature (blue), young (green) and suckling (purple) cavies in both sites—Lurhala and Kabamba represented by the continuous and dotted lines, respectively

Mean live weight of mature cavies was 758 g (with 1058 g for pregnant cavies), 441 g for young and 162 g for suckling (Fig. 4). Differences between sites were large, mainly for mature and young animals with heavier animals in Lurhala compared to Kabamba. However, weights of suckling were comparable, indicating that cavies started from a similar base, but reached a different mature weight. Farmers in Lurhala explained the weight difference by feed quantity and quality. The second reason mentioned was housing; cavies were kept indoors and in Lurhala people often lived in a ‘tapi’ (i.e. traditional round house constructed of natural materials), while in Kabamba, modern housing was more commonly found (i.e. brick walls and metal roof). The natural construction materials of Lurhala were believed to provide a more constant indoor-climate, allowing the cavies to grow better and quicker. Farmers in Kabamba attributed the difference to the animals’ race (i.e. genetics), feeding practices and the degree of farmer experience.

### Fodder

#### Quantities: offered and refused

**Fig. 5:**
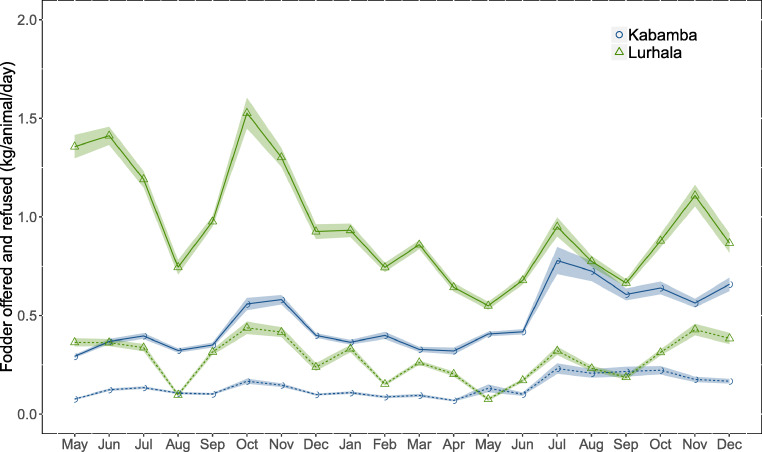
Mean quantities of fodder offered (full line) and refused (dashed line) (kg per animal per day) over time (plus and minus the standard error indicated by the shaded area)

Overall, cavies in Lurhala received more fresh fodder than cavies in Kabamba: mean daily quantities were 0.96 kg per animal in Lurhala and 0.46 kg per animal in Kabamba (Fig. 5). This difference corresponded with larger land holdings per household and the presence of communal pastures, leading to larger fodder availability in Lurhala. At the beginning of the monitoring period, fodder quantities in both sites differed clearly, but over time they converged (Fig. 5). Mean daily quantities of refusals were generally larger in Lurhala, at 0.49 kg per animal compared to 0.18 kg per animal in Kabamba. Refusals were frequently removed and used for composting. Although farmers generally regarded the dry season (i.e. July and August) as a difficult time to collect sufficient amounts of fodder, effects of seasonality on offered fodder amounts were not observed.

**Fig. 6:**
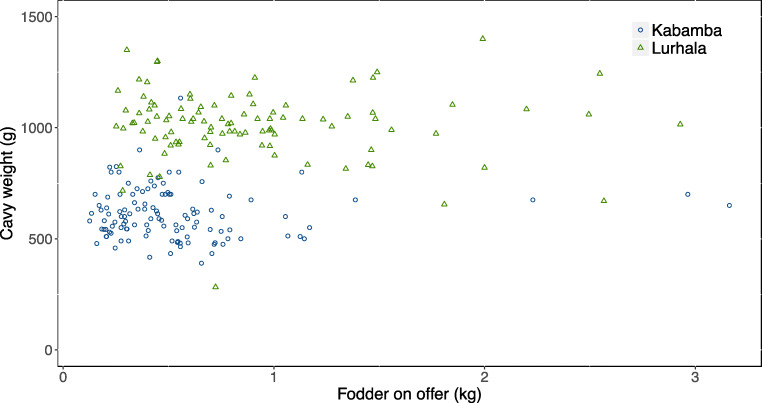
Mean live weight of mature cavies versus mean quantity of fodder on offer (data point per household per month)

Daily fodder demand of a cavy was estimated to be 5–15% of its live weight (Cicogna 2000; Lammers et al. 2009). Taking an average requirement of 10% of the live weight, and based on the measured mean live weights of 605 g and 1002 g in Kabamba and Lurhala, a cavy needed 60 g and 100 g dry matter (DM) per day, respectively. Assuming 20% DM content, fresh fodder requirement was around 300 and 500 g per cavy per day, which was less than the fodder quantity on offer in Kabamba and Lurhala (i.e. 0.46 kg and 0.96 kg). Correlation analysis indicated that cavy live weight and temporal changes therein were not related to the amount of fodder on offer (Fig. 6). Likewise, the increase in fodder on offer during the last months of the campaign in Kabamba was not matched by an increase in cavy live weight.

#### Composition of fodder offered

**Fig. 7:**
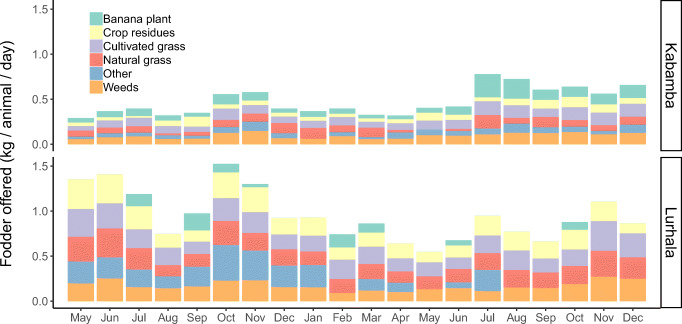
Fodder on offer over time in Kabamba (top) and Lurhala (down), stacked by six fodder groups, i.e. cultivated grass, naturally growing grass, weeds, banana plant, crop residues and ‘other’ fodders (i.e. unspecified by farmers or mixtures of leaves, crop residues and various plant parts)

During most months, crop residues, naturally-growing and cultivated grasses were the main fodder on offer to cavies, followed by weeds, collected along roads or fields (Fig. 7). Differences in fodder composition between sites were large. In Kabamba, grasses (both naturally growing and cultivated) and weeds were the main groups of fodder on offer, complemented by parts of the banana plant and crop residues. In Lurhala, crop residues together with weeds and grasses (both naturally growing and cultivated) formed the basis of the cavy diet. The composition of cavies’ diets varied over time, with parts of the banana plant and ‘other’ being major during some, but completely absent, during other months. The composition was relatively constant at Kabamba, but showed much more variation over time at Lurhala.

### Livestock sales and household consumption

The recall data from the household survey indicated very few cavy sales; less than one cavy was sold per household per year in Kabamba and slightly more than three cavies sold per household in Lurhala. In contrast with this, the on-farm records pointed to regular cavy sales, which were commonly given as an explanation for decreases in herd size. The market price of cavies was low but stable at 0.95 USD (standard deviation of 0.16 USD) to sell and 1.18 USD (standard deviation of 0.13 USD) to buy.

**Fig. 8:**
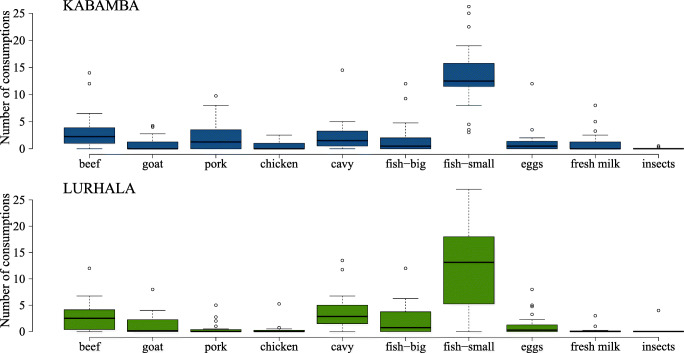
Boxplots of monthly consumptions of ten animal products per household in Kabamba and Lurhala

According to the weekly farmer records, the overall mean number of consumptions of animal products per month was 26 in Kabamba and 23 in Lurhala (Fig. 8). In both sites, small fish were the most frequently consumed, with a mean of 13 times per month. Meat was consumed 9 and 8 times per month, with 2 and 4 cavies eaten monthly per household in Kabamba and Lurhala, respectively. Cases of intra-household differentiation in cavy consumption were recorded, as cavy meat was mostly regarded as food for children. The estimated quantity of consumed cavy meat per person per month was 100–160 g in Kabamba and 220–375 g in Lurhala. Assuming a 20% protein content of cavy meat (Huss and Roca 1982), 0.8 g protein requirement for humans per kg body weight and a body weight of 30 kg and 60 kg for children and adults, the cavy meat intake provided 3–10% of the protein requirements of children and 1–5% for adults. However, if the cavy meat would preferentially be allocated to children, about 10–25% of their daily protein requirements could be met. Although this seemed little, in both sites, cavy meat was consumed nearly five times more frequently than chicken meat and made up at least one-fifth of all meat consumption instances. Especially for the poorest households, the number of times cavies were consumed was over half of the monthly number of meat consumptions.

## Discussion

Farmers in South Kivu generally owned small livestock herds that mainly consisted of small and micro-livestock and few cattle. In these small herds, cavies were important especially for the poorest households. The majority of farmers kept cavies primarily for home consumption and nearly all children and about 60% of all women and men consumed cavies (Meutchieye et al. 2013). Health benefits related to livestock ownership (Rawlins et al. 2014) are easier realized through micro-livestock compared to large livestock, because small animals with rapid growth and reproduction rates make an easy and continuous harvest possible (Lammers et al. 2009). Even the small quantities of animal-source foods recorded here could substantially increase the diet quality (Murphy and Allen 2003) by providing important micronutrients and protein, which often are not, or not sufficiently, available in smallholder farmers’ diets (Scrimshaw 2018). The second most important reason for keeping cavies was cash generation. A recent study comparing incomes in rural South Kivu found farmers’ incomes to be lowest, with 91 USD per month, against 216 USD for miners and 379 USD for agri-miners (i.e. households combining agricultural and mining activities) (Vwima et al. 2017). Considering the low farm income, cavy sales could be relatively meaningful, especially for the poorest farmers. Cavy numbers were reported to decrease strongly at the time of payment of school fees (Meutchieye et al. 2013), but this trend was not clearly visible from the collected data. Monthly fees were about 2–3 USD per child in primary school (Maass et al. 2010), resulting in a need to sell 2–3 mature cavies every month in order to keep one child in school. The mean number of cavies per household was 10, with a litter size of 2–4. Assuming 8 females, each carrying 2–3 litters per year, a mean production of 32–72 cavies per household per year could be reached, potentially generating 32–72 USD as cash income if sold, or, based on a 20% protein content (Huss and Roca 1982), 5–11 kg protein if consumed.

Generally, the findings on cavy herd size and productivity in South Kivu were within the range of the scant literature for this region (Maass et al. 2010; Meutchieye et al. 2013) but less than those reported on cavies kept under improved circumstances (Lammers et al. 2009; Cicogna 2000). Between the two study sites, clear differences in cavy live weights were observed, which could be attributed to differences in cavy husbandry (feeding and housing) and/or genetics. Whereas further research is needed to confirm the cavy genetics hypothesis, feeding practices did not offer a plausible explanation. This was concluded, firstly from the fact that in both sites, feed was not limiting, as the quantity of fodder on offer was consistently larger than cavies’ demand. Explanations for overfeeding included the relative ease to collect sufficient fodder for such small animals, the easy dirtying of the feed on the ground (i.e. no feeder), and the purposeful use of refusals for bedding. Secondly, the amount of fodder offered did not correlate with cavy live weight (Fig. 6).

Even though keeping cavies was popular among smallholders, farmers faced many challenges. Firstly, cavy herd sizes experienced frequent and large declines due to theft, predation by cats and dogs or a cow stepping on the animals (Zozo et al. 2010; Desiere et al. 2015). Secondly, farmers lacked knowledge regarding breeding. As cavies were generally not caged and only a light form of mating control was applied, inbreeding was likely, leading to animals being fertile later, with less and weaker offspring (Ngou-Ngoupayou et al. 1995; in Maass et al. 2010). Thirdly, feed-related constraints included a lack of knowledge on good feeding practices and the time needed to collect fodder during the dry season (Maass et al. 2010). Despite the fact that fodder quantity seemed not to be a problem, the large amount of refusals and the relative importance of low-quality banana fodder (Klapwijk et al. 2014), indicated that fodder quality could be a limiting factor. Furthermore, the increase in fodder on offer in one of the sites during the research campaign (Fig. 5) pointed to a change in practice based on increased knowledge and awareness about the importance of feeding, which was confirmed by farmers expressing their appreciation for record keeping.

As mortality was a main limiting factor, cages or enclosures may be a solution for farmers to maintain or increase their herd sizes. An additional benefit of confining cavies is the possibility to control breeding. To improve fodder quality and reduce collection time improved fodders could be cultivated, a practice that was not common currently (Ouma and Birachi 2011). Improved fodders need to be high-yielding, locally adapted and tolerant to drought stress and could be cultivated in niches such as field edges, road sides and on degraded (or sloping) land, in order to decrease competition with the cultivation of crops. In the context of South Kivu, Napier grass (*Pennisetum purpureum*) and *Brachiaria humidicola* (Rendle) Schweick are promising options.

The sample size of 15 participating households was relatively small, but on-farm data was collected intensively, including daily measurements over a long time period. By revealing the opportunities and limitations for micro-livestock in areas like South Kivu, this study provided a starting point for follow-up research, on e.g. cavy genetics, and on effective options to improve cavy husbandry and feeding practices that are feasible for smallholder farmers.

## Conclusions

Cavies are an important asset for many smallholder families in South Kivu, eastern DR Congo. Although cavy production was highly variable, cavies were consumed regularly. Their small size and rapid reproduction rate resulted in the ability to harvest regularly, compared to larger livestock types. The quantity of fresh fodder on offer was larger than fodder demand and bigger in Lurhala than in Kabamba, which was in line with the difference in cavy live weight between the sites. Opportunities to increase cavy production can most likely be realised through the introduction of cages, in order to limit mortality and by the cultivation of improved fodders, in order to improve fodder quality. Micro-livestock (e.g. cavies) are a good entry-point for development initiatives, also outside DR Congo, because of their potential to decrease poverty and to increase health through improved nutrition.
